# ATP Release from Vascular Endothelia Occurs Across Cx43 Hemichannels and Is Attenuated during Hypoxia

**DOI:** 10.1371/journal.pone.0002801

**Published:** 2008-07-30

**Authors:** Marion Faigle, Jessica Seessle, Stephanie Zug, Karim C. El Kasmi, Holger K. Eltzschig

**Affiliations:** 1 Department of Anesthesiology and Intensive Care Medicine, Tübingen University Hospital, Tübingen, Germany; 2 Mucosal Inflammation Program, Department of Anesthesiology and Perioperative Medicine, University of Colorado Health Science Center, Denver, Colorado, United States of America; Dresden University of Technology, Germany

## Abstract

**Background:**

Extracellular ATP is an important signaling molecule for vascular adaptation to limited oxygen availability (hypoxia). Here, we pursued the contribution of vascular endothelia to extracellular ATP release under hypoxic conditions.

**Methodology, Principal Findings:**

We gained first insight from studying ATP release from endothelia (HMEC-1) pre-exposed to hypoxia. Surprisingly, we found that ATP release was significantly attenuated following hypoxia exposure (2% oxygen, 22±3% after 48 h). In contrast, intracellular ATP was unchanged. Similarly, lactate-dehydrogenase release into the supernatants was similar between normoxic or hypoxic endothelia, suggesting that differences in lytic ATP release between normoxia or hypoxia are minimal. Next, we used pharmacological strategies to study potential mechanisms for endothelial-dependent ATP release (eg, verapamil, dipyridamole, 18-alpha-glycyrrhetinic acid, anandamide, connexin-mimetic peptides). These studies revealed that endothelial ATP release occurs – at least in part - through connexin 43 (Cx43) hemichannels. A real-time RT-PCR screen of endothelial connexin expression showed selective repression of Cx43 transcript and additional studies confirmed time-dependent Cx43 mRNA, total and surface protein repression during hypoxia. In addition, hypoxia resulted in Cx43-serine368 phosphorylation, which is known to switch Cx43 hemi-channels from an open to a closed state.

**Conclusions/Significance:**

Taken together, these studies implicate endothelial Cx43 in hypoxia-associated repression of endothelial ATP release.

## Introduction

Extracellular nucleotide and nucleoside levels are increased during conditions of limited oxygen availability (hypoxia) [1]–[17] and have been suggested in various aspects of physiological adaptation to hypoxia [1]–[3], [5]–[9], [16]–[19]. Particularly extracellular elevations of ATP are critical in vascular adaptation to hypoxia, as extracellular ATP can either signal directly to ATP receptors [20], [21] or is rapidly converted to adenosine leading to increased vascular nucleoside signaling [22]–[27]. While previous studies have shown that during hypoxic conditions, adenosine stems mainly from ATP-phosphohydrolysis [7], [8], [26], [28] cellular sources and mechanisms for extracellular ATP release during hypoxia are not well defined. As such, different cell types can contribute to elevations of vascular ATP during hypoxia, including circulating blood cells such as platelets, erythrocytes or inflammatory cells (e.g. PMN).[29] In addition, vascular endothelia may represent an important cellular source for extracellular ATP release during hypoxia [28] as they extend over a large surface area and are anatomically positioned at the interface between a hypoxic stimulus and the surrounding tissues [26].

At present, several studies have detailed different molecular mechanisms involved in cellular release of ATP into the extracellular milieu. Such mechanisms differ between multiple cell types (e.g. platelets, astrocytes, umbrella cells of the bladder) [30]. For example, ATP release from umbrella cells of the bladder is dependent on mechanical pressure stimulation. This ATP release can be blocked by inhibitors of connexin hemichannels, ABC-protein family members, or nucleoside transporters [31]. Other studies on cellular mechanisms of ATP release found that ATP release from neuronal astrocytes involves the gap-junction molecule connexin 43 (Cx43) [32]. As such, Cx43 molecules can assemble to hexadimers (so called “connexons”) that form junctional connections between different cell types. In addition to their role as gap-junctional proteins, recent studies indicate that Cx43 connexons are also active in single plasma membranes and can function in intercellular signaling as ATP release channels [33]. As such, genetic and pharmacological studies of neutrophil-dependent ATP release suggest a critical role of Cx43-hemi-channels in extracellular ATP release following inflammatory stimulation [29], which may play an important role in purinergic chemotaxis [34], [35]. Similarly, a very recent study studied the contribution of Cx43 to ovarian follicle development in the mouse. The authors found that Cx43 is strongly expressed in granulosa cells, in which it forms intercellular gap junction channels that couple the cells metabolically. However, recent evidence indicates that undocked gap junction hemichannels can also have physiological roles such as mediating the release of small messenger molecules, including ATP. In this study, the presence of undocked Cx43 hemichannels in granulosa cells was revealed by dye uptake induced either by mechanical stimulation or by the reduction of extracellular divalent cations, both of which are known triggers for hemichannel opening. ATP release was also detected, and could be abolished by connexin-channel blockers [36].

In the present study, we tested the hypothesis that vascular endothelia represent an important source for extracellular ATP elevation during hypoxic conditions. To our surprise, we found that extracellular ATP release from vascular endothelia is attenuated following hypoxia exposure. Additional studies to address molecular mechanisms of endothelial-dependent ATP release suggested a functional contribution of Cx43 in endothelial ATP release. Moreover, hypoxia exposure of endothelia revealed that Cx43 expression is attenuated by hypoxia, while Cx43-Serine368 phosphorylation status is increased, which has previously been shown to change the Cx43 channels from an open to a closed status [37]. These findings suggest that extracellular elevations of vascular ATP levels during hypoxia predominantly stem from other cell types than vascular endothelia (e.g. inflammatory cells, or red blood cells) [29], [35], [38], [39].

## Materials and Methods

### Endothelial Cell Culture

Human microvascular endothelial cells (HMEC-1) were a kind gift of Francisco Candal, Centers for Disease Control, Atlanta, GA [40], [41]. These cells are derived from dermal tissues and were harvested and cultured by a modification of methods described previously [9], [23]–[25], [29], [42]. In brief, HMEC-1 were harvested with 0.1% trypsin and incubated at 37°C in 95% air/5% CO_2_. Culture medium was supplemented with heat-inactivated fetal bovine serum, penicillin, streptomycin, L-glutamine, epidermal growth factor, and hydrocortisone. In other studies, human umbilical vein endothelial cells (HUVECs) were cultured as described previously [26], [43]. All experiments were carried out when cultured endothelial cells were fully confluent.

### Measurement of extracellular ATP release into the supernatant from HMEC-1

Confluent HMEC-1 monolayers were exposed to normoxia or indicated time periods of hypoxia (2% oxygen). These studies were carried out in the presence of the polyoxotungstate Na_6_[H_2_W_12_O_40_] (POM-1, 100 µM), a highly potent non-specific inhibitor of ecto-nucleoside triphosphate diphosphohydrolases [7]. The culture medium was removed, cells were washed twice and incubated as indicated in calcium containing HBSS, and samples from the supernatant were collected, shock frozen and stored at −80°C for further analysis. In subsets of experiments, intracellular ATP levels were measured. Here, confluent HMEC-1 monolayers were exposed to hypotonic lysis by addition of ice-cold water. Following centrifugation over for 5 min at 13,000 rpm, the ATP content in the supernatants was quantified using a highly sensitive luciferase based technique (CHRONO-LUME, Chrono-Log Corp., USA, Haverton). Luciferase activity was measured on a luminometer (Turner Designs Inc., Sunnyvale, California, USA) and compared with ATP standards [26], [29].

### Measurment of lactate dehydrogenase (LDH) in supernatants from HMEC-1

To assess lytic ATP release, the cytotoxicity detection kit (Roche Diagnostics, Germany, Mannheim) was used according to the manufacture's instructions.[29] In short, confluent HMEC-1 monolayers were exposed to normoxic or hypoxic (2% oxygen) conditions over indicated time periods, then 100 µl of the substrate mixture from the kit were added to 100 µl of the collected protein-free supernatant. After an incubation period of 30 min the absorbance was measured at 490 nm. In control studies, normoxic HMEC-1 were treated with 2% Triton X-100 to assess maximal LDH release.

### Pharmacological studies on mechanisms of endothelial-dependent ATP release

Confluent HMEC-1 were seeded on 6-well plates, culture medium was removed and cells were washed with calcium free 5 M HEPES HBSS. In subsets of experiments, HMEC-1 monolayers were pretreated over 10 min with 18-alpha-glycyrrhetinic acid (18αGA; Sigma Aldrich, 20 µmol), Anandamide (Sigma Aldrich, 40 µmol) or the phosphokinase C inhibitor bisindolylmaleimide (BIM, Sigma Aldrich, 10 µM). Furthermore, the non specific ABC receptor antagonist Verapamil (Tocris Cookson, 10 µmol) and the inhibitor of equilibrative nucleoside transporters dipyridamole (Sigma Aldrich, 10 µmol) were used. In other studies, the effects of connexin mimetic peptides for connexin 43 (SRPTEKTIFII, Biosource, Germany, Solingen, 50 µmol) and connexin 40 (SRPTEKNVFIV, Biosource, Germany, Solingen, 50 µmol) were used (100 µM). After 30 min of incubation time at room temperature, ATP content within the supernatants was measured as described above. Connexin mimetic peptides that correspond to short specific sequences in the two extracellular loops of connexins are a class of benign, specific and reversible inhibitors of gap-junctional communication that have been studied recently in a broad range of cells, tissues and organs. The properties and uses of these short synthetic peptides, and their probable mechanism of action with those of a wide range of other less specific traditional gap-junction inhibitors have been recently reviewed [44].

### Transcriptional analysis

To assess the influence of hypoxia on enthothelial connexin transcript levels, HMEC-1 monolayers were exposed to hypoxia (2% oxygen) over indicated time periods, followed by isolation of RNA and quantification of transcript levels by real-time RT-PCR (iCycler; Bio-Rad Laboratories Inc.). RNA was isolated by using Total RNA purification NucleoSpin RNA II according to the manufacturer's instructions (Marcherey-Nagel, Germany, Düren). RNA concentration was measured followed by cDNA synthesis using the iScript cDNA Synthesis Kit (Bio-Rad Laboratories). The PCR reactions contained 10 pM sense and 10 pM antisense oligonucleotides with iQ SYBR Green Supermix (Bio-Rad Laboratories). The target sequence was amplified using increasing numbers of cycles of 95°C for 15 s, 56°C for 30 s, 72°C for 15 s as described previously [26], [42]. Realtime RT-PCR conditions and primer sequences are summarized in Table 1.

**Table 1:** Human primer pairs used for real-time RT-PCR.

Human	Forward (5′–3′)	Reverse (5′–3′)	Product [bp]	Temp [C°]
ß-actin	GGTGGCTTTTAGGATGGCAAG	ACTGGAACGGTGAAGGTGACAG	162	56
Cx 25	CAGGCCTCTTGCCGATTCAG	GTGGCCTCCACTTCCTATCA	215	56
Cx 26	AAGAAGTCGCTTGGGAATTT	GCTGAAGGGGTAAGCAAACA	177	56
Cx 30	TCCAGAAGGCAATACCAACC	CAATGCTCC TTTGTCAAGCA	180	56
Cx 30.3	AGAGGTGCATGGAGATCTTC	CAGCCTTCATTAGGACAGAG	135	56
Cx 31	CTTCCAGCAGCAGCAGGTCT	CACCAGCCTGAGCACAGTTG	181	56
Cx 31.1	ATCTACCTGGTGAGCAAGAG	GAGAGGAGGATGACTGTCTG	162	56
Cx 31.3	GAGAACCTTGCCTTGGTAGT	TTGTGTCTTCTGGTGCTCTC	214	56
Cx 31.9	GACCGTCTTCGTGCTCTTCT	AGCAGCTTCTGCGCCTCTTC	153	56
Cx 32	TCCGACAGCGTCTCCAATTA	TTGTGGCCAGCAAGCACTAT	187	56
Cx 36	GCATCAAGGAGGTGGAATGT	TTGAGTTCAGCCAGGTTGAG	112	56
Cx 37	CAACAGAGGGGTCCTGAGAA	CTGGAGAGGAAGCCGTAGTG	182	56
Cx 40	AGCAGGGGCAAGGAAATAGT	TACAGAGACCAGGCCAATCC	162	56
Cx 40.1	GCCGTCTTCAGCGTCTATGT	GAGGAGGAGGTGGATGATGT	180	56
Cx 43	AATTCAGACAAGGCCCACAG	CATGGCTTGATTCCCTGACT	216	56
Cx 45	ACGCTTGGATCTGGCAGTTC	TCAGTGAGCTGCTGCTTACC	209	56
Cx 46	TTCGAGCTGAAGCCGCTCTA	CGCCAGCATGAAGATGATGA	111	56
Cx 47	GACCACCGTGTGGATCTGAG	CGGCTAAGGAGAAGGCTGAG	113	56
Cx 50	CTCCACTCCATTGCTGTCTC	CGTAGGAAGGCAGTGTCTCT	217	56
Cx 59	AAGAGACCACAGCCTTAGGA	AGGAGTCCAGTCTAGAAGGA	118	56
Cx 60	CAGTGAAGGCAGCATGAGAG	TGACTGAAGGCAGAGGTGAG	197	56

### Western blot analysis

Cx43 expression and phosphorylation status were assessed by Western blot analysis using connexin 43 rabbit polyclonal antibody (Cell Signaling) or phospho-connexin 43 rabbit polyclonal antibody detecting serine 368 phosphorylated Cx43 (Cell Signaling) as described previously.[29] In subsets of experiments, HMEC-1 were pre-treated with the phosphokinase C (PKC) inhibitor bisindolymaleimid during hypoxia or normoxia exposure (10 µM) [45].

### Immunoprecipitation

Surface Cx43 expression was assessed as described previously.[29] In short, confluent HMEC-1 were exposed to normoxia or indicated time periods of hypoxia (2% oxygen). Monolayers were washed, surface proteins labeled with biotin, lysed and centrifugated to remove cell debris. Protein concentration was measured and immunoprecipitation was performed with 2 µg monoclonal rabbit anti-mouse connexin 43 antibody (Acris) and incubated overnight. Subsequently, 50 µl Protein G Microbeads (Miltenyi Biotec) were added and incubated for 30 minutes on ice. Magnetically separated immune complexes were eluated with 1× SDS gel loading buffer, transferred to nitrocellulose, and blocked overnight in washing buffer with 3% BSA and 2 µg/ml streptavidine. Membranes were enhanced by alkaline phosphatase.

### Data analysis

Data were compared by two-factor ANOVA, or by Student's *t* test where appropriate. Values are expressed as the mean±SD from at least three separate experiments. Standard deviation error bars at baseline (time point 0) reflect differences between different experiments.

## Results

### Extracellular ATP release from vascular endothelia is attenuated by hypoxia

**Figure 1:**
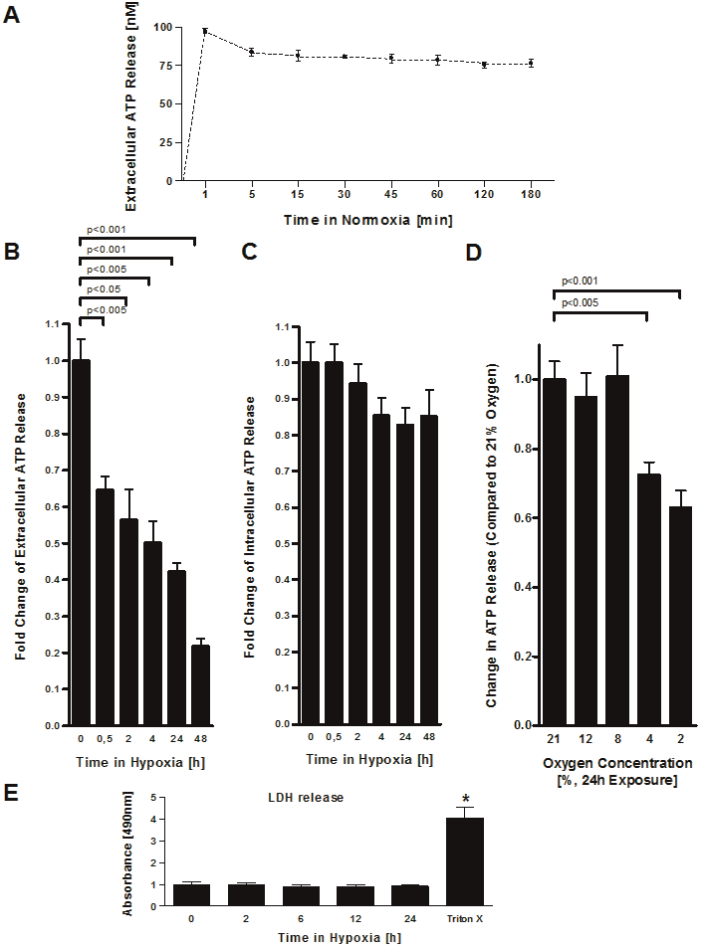
Endothelial ATP release during hypoxia. A, To study extracellular ATP release from normoxic endothelia, monolayers of confluent HMEC-1 were washed and the culture media was replace with calcium containing HBSS. ATP content from their supernatant was sampled at indicated time points and quantified using a luminometric ATP detection assay. B, To measure extracellular ATP release under hypoxic conditions, confluent HMEC-1 monolayers were exposed to hypoxia (2% oxygen) over indicated time periods. Culture media was replaced with calcium containing HBSS and the ATP content within the supernatant was measured after 30 min incubation time. C, For intracellular ATP measurement, confluent HMEC-1 monolayers were exposed to hypoxia over indicated time periods, culture medium was discarded and cells were lysed by adding ice-cold water. ATP concentrations were measured as above. D, To measure the influence of different oxygen concentrations on endothelial ATP release, HMEC-1 were exposed to indicated degrees of hypoxia (21–2% of oxygen) over 24 h. Culture media was replaced with calcium containing HBSS and the ATP content within the supernatant was measured after 30 min incubation time. E, Confluent HMEC-1 monolayers were exposed to hypoxia as indicated. To assess lytic ATP release, LDH concentrations within the supernatant were measured by an LDH detection kit. In control experiments, cells were lysed with Triton X-100 (*p<0.01, n = 6 for all experiments).

To study the role of vascular endothelia in extracellular ATP release, we first measured the extracellular ATP content within the supernatant of vascular endothelia. For this purpose, the culture media from confluent HMEC-1 monolayers was replaced with calcium containing HBSS, and samples from the supernatant were collected at indicated time points, in the presence of POM-1, a highly potent ENTPDase inhibitor [6], [7]. As shown in Figure 1A, ATP levels within the supernatant immediately rise to approximately 90 nM and remained stable over the examined time period of 180 min. The present results are consistent with other studies that have measured ATP release from endothelial cells or fibroblasts. In these studies, the authors found maximal ATP levels at 10 min (their earliest measurement point, fibroblasts) or 30 min (endothelia) and no additional elevation at later points (e.g. at 60 min or 24 h). In fact, ATP levels were attenuated at these later measurement points. To rule out acute effects of changing the media (e.g. cell lysis initiated by media change), all further measurements were carried out 30 min after changing the media.

To study the influence of hypoxia on vascular ATP release, we pre-exposed over vascular endothelia (HMEC-1) over indicated periods to normoxia (21% oxygen) or ambient hypoxia (2% oxygen), the media was replaced and ATP content was measured within the supernatants after 30 min of incubation time. Surprisingly, we found that extracellular ATP levels were lower in supernatants derived from post-hypoxic endothelia in a time-dose dependent fashion, suggesting that endothelial ATP release is attenuated by hypoxia (Figure 1B). As next step, we lysed cells following different time-periods of hypoxia exposure and measured intracellular ATP levels. As shown in Figure 1C, intracellular ATP levels were unchanged in post-hypoxic endothelia. This is important, as changes in extracellular ATP release with hypoxia as shown in Figure 1B could be caused by attenuated intracellular ATP content, thereby resulting in an overall decrease in the transcellular ATP gradient during hypoxia. To study the degree of hypoxia that is necessary for attenuating endothelial ATP release, we next performed an oxygen-concentration dose-response curve with oxygen concentrations spanning the physiological range from 0 to 100 mmHg. For this purpose we exposed cultured endothelial cells (HMEC-1) over 24 h to indicated concentrations of oxygen, ranging from normoxia (21% oxygen) to severe hypoxia (2% oxygen). As shown in Figure 1D, attenuated ATP release occurred only with more severe hypoxia (4% or 2% oxygen over 24 h). To study the contribution of lytic ATP release, we measured LDH content in the supernatants of post-hypoxic endothelia. As shown in Figure 1F, LDH content in the supernatant (obtained 30 min after changing the media) did not change significantly with hypoxia exposure, suggesting that lytic ATP release did not account for differences between normoxic or post-hypoxic endothelial ATP release. Taken together, these studies demonstrate that ambient hypoxia of vascular endothelia results in decreased extracellular ATP release.

### Mechanisms of endothelial ATP release

**Figure 2:**
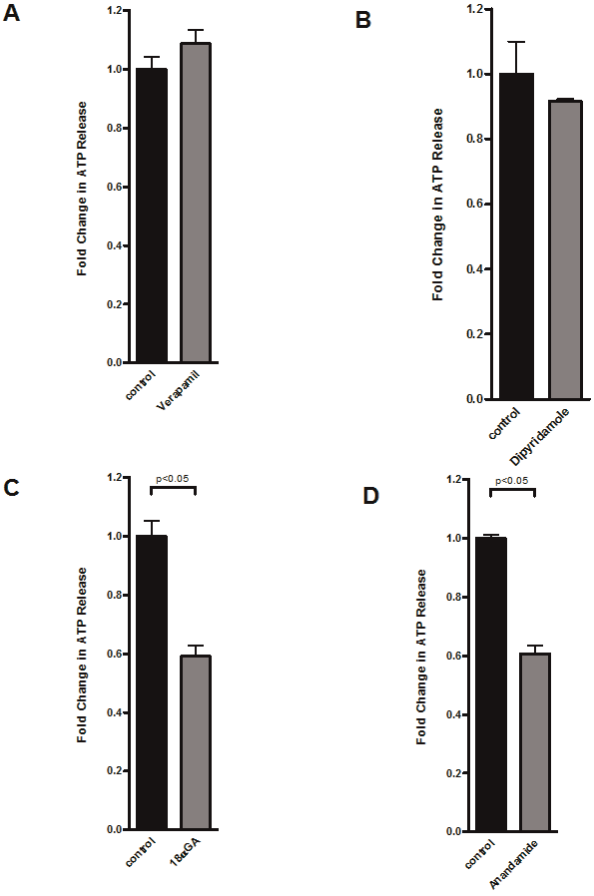
Molecular mechanisms of endothelial-dependent ATP release. A–D, Confluent HMEC-1 monolayers were washed and exposed to Verapamil (10 µmol) or Dipyridamole (1 µmol) over 20 min, 18-alpha-glycyrrhetinic acid (18αGA, 20 µmol) or Anandamide (40 µmol) over 10 min. ATP content within the supernatant was measured by a luminometric ATP detection assay and compared with untreated control cells (n = 6).

**Figure 3:**
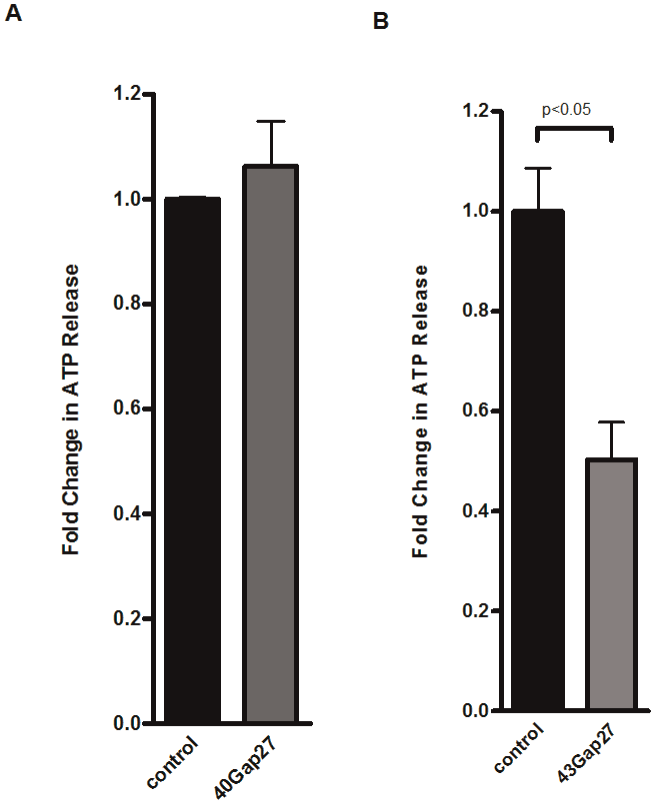
Connexin-mimetic peptides in endothelial ATP release. A, B, Confluent HMEC-1 monolayers were washed and treated with connexin-mimetic peptides (A: Cx40 peptide, SRPTEKNVFIV, 50 µmol; B: Cx43 peptide, SRPTEKTIFII, 50 µmol). ATP content within the supernatant was measured by a luminometric ATP detection assay after an incubation period of 20 min and compared with control HMEC-1 treated with 50 µM bovine albumin (n = 6).

ATP exists in the cytoplasm at millimolar concentrations[46] and can be released extracellularly by several mechanisms, including transport via connexin hemichannels, through nucleoside transporters, or direct transport through ATP-binding cassette (ABC) proteins.[29] As first step, we tested the effect of verapamil, an inhibitor of several ABC proteins and the multi drug resistance gene product.[29] As shown in Figure 2A, we found no alteration in endothelial ATP release with verapamil treatment. Similarly, the nucleoside transport inhibitor dipyridamole had no effect on endotheilal ATP release (Figure 2B). Next, we measured ATP release of endothelia in the presence or absence of the non-specific gap junction inhibitor 18αGA.[29] As shown in Figure 2C, addition of 18αGA resulted in a dramatic reduction of ATP release from normoxic endothelia. Similarly, treatment with the non-specific gap junction inhibitor anandamide [29] resulted in attenuated ATP release (Figure 2D). We extended these findings to define specific connexin contributions to endothelial ATP release. For these purposes, we used previously described connexin mimetic peptides specifically directed against Cx40 or Cx43 [29], [47]. As shown in Figure 3A, the Cx40-specific connexin mimetic peptide did not alter ATP liberation from endothelia. By contrast, the peptides which block Cx43 showed a significant inhibition of ATP liberation (50.0±7% reduction, p<0.01 by ANOVA, Figure 3B). These results implicate Cx43 in ATP release from human endothelia.

### Modulation of endothelial connexin expression by hypoxia

**Figure 4:**
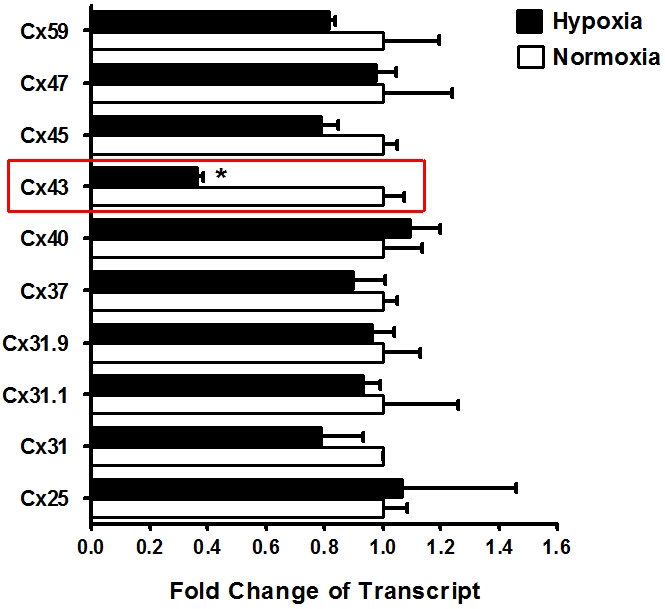
Endothelial connexin expression. Confluent HMEC-1 monolayers were exposed to normoxia or hypoxia (12 h). Total RNA was isolated and real-time reverse-transcriptase polymerase chain reaction was employed to screen for transcriptional modulation of connexin expression. Data were calculated relative to an internal control gene (β-actin) and are expressed as fold change over normoxia at each time point. Results are derived from 3 experiments in each condition.

**Figure 5:**
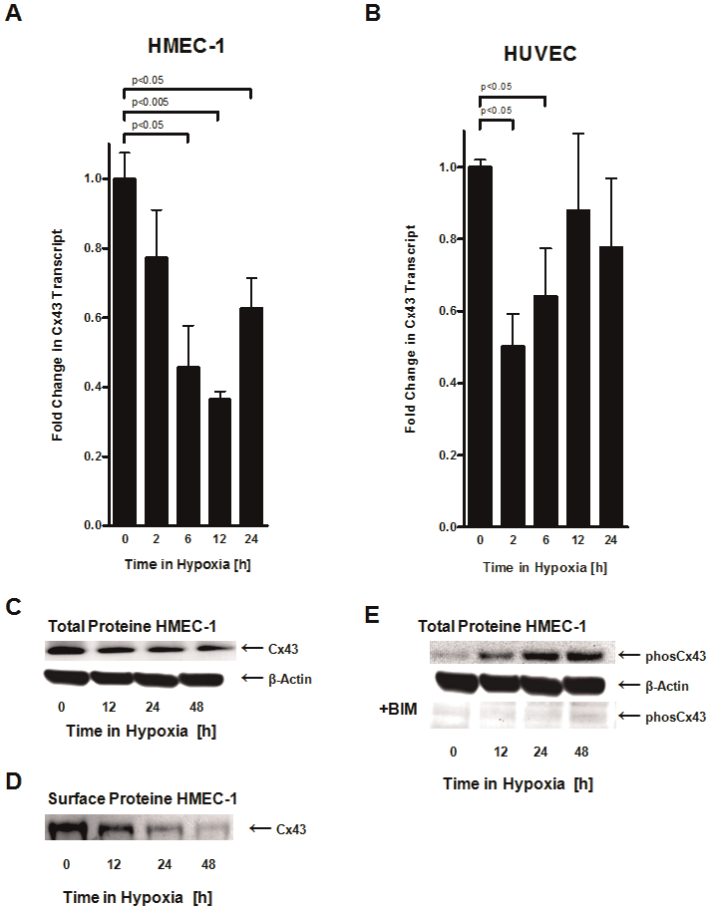
Influence of hypoxia on Connexin 43 expression. A, B. Confluent HMEC-1 or HUVEC monolayers were exposed to normoxia or hypoxia (2% oxygen) over indicated time periods. Total RNA was isolated and transcriptional responses were assessed by real-time reverse-transcriptase polymerase chain reaction. Data were calculated relative to an internal housekeeping gene (β-actin) and are expressed as fold change over normoxia at each time point. Results are derived from 3 experiments in each condition. C. Confluent HMEC-1 monolayers were exposed to hypoxia over indicated time periods. Cells were lysed and proteins were resolved by SDS-PAGE and transferred to PVDF-membrane. Membranes were probed with a connexin 43 antibody, proteins were detected by chemiluminescene. The same blot was reprobed for β-actin as a control for protein loading. A representative experiment of 3 is shown. D. Confluent HMEC-1 monolayers were exposed to hypoxia over indicated time periods. Monolayers were washed, surface proteins were biotinylated, and cells were lysed. Connexin 43 was immunoprecipitated, followed by addition of Protein G Microbeads. Proteins were resolved by SDS-PAGE and resultant Western blots were probed with Streptavidin. A representative experiment of 3 is shown. E. Confluent HMEC-1 monolayers were exposed to normoxia or hypoxia over indicated time periods. Cells were lysed and proteins were resolved by SDS-PAGE and transferred to PVDF-Membrane. Membranes were probed with phospho-connexin 43 antibody specific for phosphorylated ser368, and proteins were detected by chemiluminescene. The same blot was probed for β-actin as a control for protein loading. A representative of 3 is shown. In subsets of experiments, cells were pretreated with the protein kinase C inhibitor bisindolylmaleimide (10 µM; +BIM).

Based on our observations of attenuated ATP release by endothelia following hypoxia exposure and our data showing that gap-junctional inhibition is associated with attenuated endothelial ATP release, we next studied transcriptional consequences of hypoxia on endothelial connexin expression. Here, we first performed a transcriptional screen of known connexin molecules[48] using realtime RT-PCR to compare normoxic or hypoxic connexin expression of HMEC-1 (Table 1). Serendipitously, these studies revealed a selective repression of Cx43 transcript levels with hypoxia exposure (Figure 4, 2% oxygen, 12 h of hypoxia), while transcript levels of other connexins were not observed. As shown in Figure 5A, these results could be confirmed with different time periods of hypoxia exposure (Figure 5A) and in a different human endothelial cell line (HMEC-1 and HUVECs, Figure 5A and B). The percentage of Cx43 repression and kinetics are slightly different between both cell types. However, the overall observation of significant repression of Cx43 expression with hypoxia is consistent between both cell lines. The reasons for these differences between HUCECs and HMEC-1 remain unclear to the authors. Western blot analysis showed time-dependent repression of Cx43 protein (Figure 5C) and immunoprecipitation localized attenuated Cx43 protein levels to the cell surface (Figure 5D). As previous studies had shown that PKC-dependent phosphoylation of the serin368 residue of Cx43 is associated with a functional change of Cx43 channels from the open to the closed state [37], [49], we next studied Cx43-serine368 phosphorylation status by Western blot analysis using a serin368 phospho-specific antibody for Cx43. As shown in Figure 5E, hypoxia was also associated with increased phosphorylation of Cx43-Serine368. Consistent with previous reports, this response could be attenuated with the PKC inhibitor BIM (no changes in beta actin were observed, data not shown) [37], [49]. Taken together these studies reveal hypoxia-dependent repression of Cx43 transcript in conjunction with increased phosphorylation of Cx43-Serine368, suggesting that hypoxia attenuates endothelial ATP release by transcriptional repression and phosphorylation of Cx43.

### Functional consequences of Cx43 phosphorylation on endothelial ATP release

**Figure 6:**
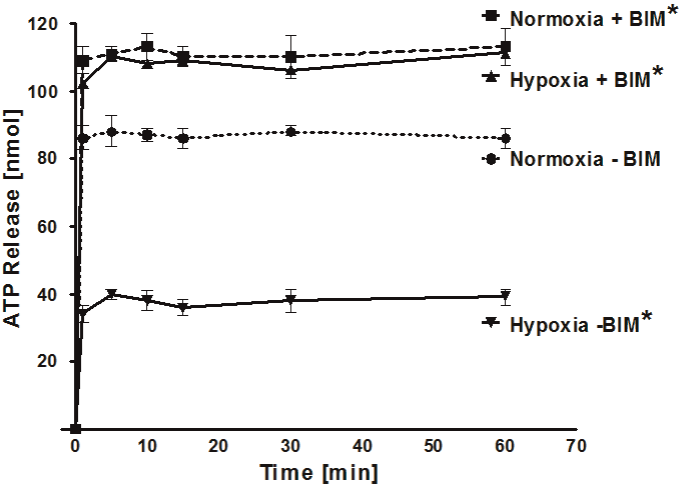
ATP release from HMEC treated with the protein kinase C inhibitor bisindolylmaleimide (BIM). To study the role of Cx43 ser398 phosphorylation status in ATP release from endothelia, monolayers of confluent HMEC-1 were treated with BIM (+BIM, 10 µM) or vehicle control (-BIM), exposed to normoxia or hypoxia (24 h, 2% oxygen), washed and the culture media was replace with calcium containing HBSS. ATP content from the supernatant was sampled at indicated time points and quantified using a luminometric ATP detection assay (*p<0.05 compared to Normoxia – BIM; n = 6).

We next pursued studies to address whether hypoxia-associated phosphorylation of Cx43 contributes to attenuated ATP release from vascular endothelia. Here, we measured endothelial ATP release in following PKC inhibition with BIM to attenuate Cx43-Serine368 (see above) under normoxic or hypoxic conditions (2% oxygen over 24 h). These studies revealed that already under normoxic conditions ATP release was significantly increased following PKC inhibition (Figure 6A) as compared to untreated normoxic HMEC-1 (p<0.05). While hypoxia exposure of untreated HMEC-1 was associated with a significant attenuation of ATP release (p<0.001), this response was completed abolished following PKC inhibition with BIM. In fact, BIM treated HMEC-1 had higher ATP levels within the supernatant than untreated normoxic HMEC-1 (p<0.05). Taken together, these data suggest a functional contribution of Cx43 phosphorylation in attenuating endothelial ATP release during conditions of hypoxia.

## Discussion

The present studies address the contribution of vascular endothelia to extracellular ATP elevations during conditions of hypoxia. While extracellular ATP levels are generally elevated during limited oxygen availability,[28] the results from our studies point out that endothelial ATP release is actually attenuated by hypoxia exposure. These findings suggest that other cellular sources (e.g. platelets, red blood cells or inflammatory cells) [29], [35], [38], [39] could account for extracellular ATP elevations during limited oxygen availability or provide a “trigger” for other cells to release ATP. In addition, the present studies address molecular mechanisms involved in endothelial ATP release. Consistent with previous studies suggesting a potential role of endothelial Cx43 hemichannels in extracellular ATP release [47], the present studies point towards a critical role of Cx43 in endothelial-dependent ATP release. Moreover, endothelial hypoxia results in transcriptional repression of Cx43 in conjunction with increased phosphorylation status of Cx43- ser368, thereby resulting in hypoxia-associated attenuation of ATP-release.

In contrast to the present studies, a previous study of endothelial ATP release during hypoxia found increased extracellular ATP release from pulmonary endothelia following hypoxia exposure [28]. Why these results are different from the present studies is currently unclear. A possible explanation may involve the fact, that pulmonary endothelia that were used in the studies by Gerasimovskaya et al. may be different in their responses to hypoxia than vascular endothelial cells that were used in the present studies (e.g. HMEC-1). As such, baseline levels of ATP release measured in the present studies of HMEC-1 were close to 100 nM, whereas baseline ATP levels measured by Gerasimoskaya et al. in pulmonary endothelia were approximately 0.2 nM. Moreover, differences in the dynamics of extracellular ATP metabolism in different cellular models or hypoxic conditions could also contribute to the differences between both studies. In addition, one has tokeep in mind that both studies did not really measure a release rate but an instantaneous concentration that will be the result of a omplex mixture of cell volume, media volume, and degredation rates compared to release rates, thus making it difficult to compare both experimental settings.

The findings from the present studies highlight the role of extracellular ATP release as part of a crosstalk pathway between different cell-types during conditions of hypoxia. The fact that endothelial ATP release is attenuated following hypoxia exposure suggests that other cellular components of the vasculature (e.g. smooth muscle cells, fibroblasts, platelets, red blood cells or inflammatory cells) [29], [35], [38], [39] have to account for hypoxia-associated increase in extracellular ATP. For example, non-resident cells like PMN that participate in an endothelial-neutrophil dependent crosstalk may therefore represent an important source of extracelluar ATP [25]. In fact, extracellular ATP can be rapidly metabolized to adenosine by hypoxia-induced ectonucleotidases (CD39, ecto-apyrase, conversion of ATP to AMP and CD73, ecto-5′-nucleotidase, AMP to adenosine) expressed on the endothelial surface, thereby contributing to endothelial adaptation to hypoxia. In fact, different studies have shown that during conditions of hypoxia or ischemia, the main source of extracellular adenosine stems from phosphohydrolysis of precursor molecules, particularly ATP [5]–[8], [26]. For example studies in mice deficient in extracellular adenosine generation (*cd73^−/−^* or *cd39^−/−^* mice) [26], [50] revealed dramatic increases in vascular leakage and pulmonary edema when mice were exposed to ambient hypoxia (8% of oxygen over 4 h) [26], [50]. In addition, a recent study of vascular responses to hypoxia compared gene-targeted mice for each individual adenosine receptor (AR, A1AR, A2AAR, A2BAR or A3AR) showing a barrier-protective role of signaling through the A2BAR in attenuating vascular leakage during hypoxia [27]. Moreover, these studies found resuscitation of endothelial barrier defects associated with hypoxia by A2BAR agonist treatment. While our results suggest that the general elevation of ATP levels observed in hypoxia may emanate from other cellular sources, it is important to point out that erythrocytes and platelets lack connexins [51], and thus Cx hemichannels detract from the importance of such channels in enucleate cells. Taken together, other cellular sources than endothelia and other molecular mechanisms than Cx43-dependent ATP release are likely to contribute to elevating extracellular ATP levels with hypoxia.

Some limitations of the present studies need to be pointed out. The essence of this paper is that reduced release of ATP is atypical. However, a cultured endothelial cell line is not strictly “endothelium”. These cells in a blood vessel interact extensively and multidimensionally with each other and also with smooth muscle cells and thus it is, after all, a model. In addition, it is may be surprising to see on the one hand a very strong effect of PKC inhibition (Fig 6) and on the other hand a downregulation (Fig. 5) of Cx43. For example, if Cx43 is downregulated, how can it be assumed that PKC inhibition prevents the ATP release? However, similar observations in other models have been made previously. For example, hypoxia is associated with transcriptional repression of equilibrative nucleoside transporters, particularly ENT1 thereby resulting in attenuated adenosine uptake from the extracellular space and enhanced adenosine signaling effects [22], [23]. However, pharmacological inhibition of nucleoside transporters can be used therapeutically. In this study, treatment with the nucleoside transport inhibitor dipyridamole was associated with attenuated vascular leakage during ambient hypoxia exposure of mice (8% oxygen, 4 h exposure time) [23].

Taken together, the present studies define an important contribution of endothelial Cx43 to extracellular ATP release and reveal transcription and phosphorylation dependent attenuation of extracellular ATP release from vascular endothelia during conditions of limited oxygen availability.
